# Influence of Lifestyle Parameters – Dietary Habit, Chronic Stress and Environmental Factors, Jobs – on the Human Health in Relation to the COVID-19 Pandemic

**DOI:** 10.1017/dmp.2020.222

**Published:** 2020-06-25

**Authors:** Duygu Aydemir, Nuriye Nuray Ulusu

**Affiliations:** Koc University, School of Medicine, Rumelifeneri Yolu, Sariyer, Istanbul, Turkey; Koc University Research Center for Translational Medicine (KUTTAM), Sariyer, 34450, Istanbul, Turkey

**Keywords:** COVID-19, pandemic, lifestyle

The coronavirus disease (COVID-19) has become a major public health problem since the beginning of 2020. The elderly population and people with chronic diseases are categorized as the major risk groups because of the weakened immune system compared with healthy individuals.^1^ A strong immune system is formed by our lifestyle parameters and environmental factors such as dietary habits, smoking, physical activity, chronic stress, air pollution, population growth, socioeconomic status, and industrialization. The immune system develops by age, nutrition, and antigen stimulation after birth and among them, nutrition plays a key role in the immunomodulation. Dietary habit is affected by several factors such as socioeconomic status, cultural traditions, employment, and habits, including smoking and alcohol. Being exposed to the poor socioeconomic status causes imbalanced nutrition in the individuals that impair the immune system. On the other hand, smoking, excessive alcohol consumption, and physical inactivity are known to contribute to the adverse effects on the immune system of humans.^2^

Environmental factors, including air pollution, environmental pollution, rapid industrialization, and smoking, have adverse effects on the respiratory system of the individuals, besides their negative impact on the immune system. Therefore, people with smoking habits or living close to the industrial zones may become more vulnerable to the COVID-19 infection. Extra precautions can be taken in the epidemic areas with high levels of air pollution or fields of work with high possibility of lung damage, such as mining.^3^

Additionally, chronic stress is one of the major negative effects that is brought by the demands of modern life into our lives. Persistent exposure to chronic stress impairs the immune system, endocrine system, and behavioral responses. Currently, millions of people are under stress because of COVID-19 and its consequences, such as economic crisis, unemployment, debts, and restricted social life.^4^

Essential jobs have become another parameter affecting human health during the COVID-19 pandemic. Millions of people are not able to apply self-isolation or protections for themselves, because they have to work in different fields, including markets, cargo companies, banks, mine workers, farmers, public transportation drivers, sanitation workers, health workers, and construction workers. Thus, employment is also another parameter that should be considered as a factor during the COVID-19 pandemic.^5^

In conclusion, this pandemic reminds everyone of the importance of strengthening the immune system and the adverse health effects of the global changes on public health. People with bad dietary habits, living close to industrial zones, having risky jobs, having a chronic disease, smoking, or experiencing chronic stress should take extra precautions to avoid a weakened immune system.

## References

[1] Aydemir D , Ulusu NN. Commentary: is glucose-6-phosphate dehydrogenase enzyme deficiency a factor in coronavirus-19 (COVID-19) infections and deaths? Pathog Glob Health. 2020;114(3):109-110. doi: 10.1080/20477724.2020.1751388. 32286926 PMC7241485

[2] Buonomo E , Moramarco S , Tappa A , et al. Access to health care, nutrition and dietary habits among school-age children living in socio-economic inequality contexts: results from the “For Good: Sport is Well-Being” programme. Int J Food Sci Nutr. 2019;21:1-10. doi: 10.1080/09637486.2019.1655714. 31433671

[3] Mariotti A. The effects of chronic stress on health: new insights into the molecular mechanisms of brain-body communication. Future Sci OA. 2015;1(3):FSO23. doi: 10.4155/fso.15.21. PMC5137920 28031896

[4] Haahtela T , von Hertzen L , Anto JM , et al. Helsinki by nature: the nature step to respiratory health. Clin Transl Allergy. 2019;9:57. 31695865 10.1186/s13601-019-0295-2 PMC6822361

[5] World Health Organization. Novel coronavirus (2019-nCoV). Situation report – 77. 2019 https://www.who.int/docs/default-source/coronaviruse/situation-reports/20200301-sitrep-41-covid-19.pdf?sfvrsn=6768306d_2. Accessed March 30, 2020.

